# Orthopedic Skin Closure in South India: Sutures Versus Staples and Their Postoperative Outcomes

**DOI:** 10.7759/cureus.69436

**Published:** 2024-09-15

**Authors:** Silambarasi Nagasamy, Pradeep Elangovan, Haemanath Pandian, Sheik Tajudeen, A Ganesh

**Affiliations:** 1 Orthopaedics, Chettinad Hospital and Research Institute, Chennai, IND

**Keywords:** infection, staples, suture, sutures, wound closure

## Abstract

Introduction

Skin closure is an essential step in deciding the success of a surgery. Despite this, the choice of skin closure material is frequently overlooked when making surgical decisions. Skin closure plays an important role in promoting rapid wound healing and decreasing the length of hospital stay, especially in orthopedic surgeries. Our study focused on comparing the outcome of wound closure in orthopedic surgeries with sutures and staples in a South Indian population.

Materials and methods

We enrolled 120 patients undergoing orthopedic procedures at Chettinad Hospital and Research Institute from July 2023 to February 2024. Patients were randomly assigned to skin closure with either staples or sutures. Wound inspection was done on the 2^nd^, 5^th^, 7^th^ and 12^th^ post-operative days. The patients were evaluated at six months using the Visual Analog Scale (VAS) and Hollander Wound Evaluation Score (HWES).

Results

Staple closure significantly reduced mean closure time (5.2 ± 0.9 minutes) compared to sutures (12.6 ± 1.5 minutes, p < 0.0001). Pain during staple removal was less (VAS score 1.41 ± 0.15) than with suture removal (VAS score 1.57 ± 0.2, p = 0.024). Although patient satisfaction was similar between the groups (Staples: 8.35 ± 0.45 vs. Sutures: 8.0 ± 0.5, p = 0.062), staples provided superior cosmetic outcomes (HWES: 4.1 ± 0.7) compared to sutures (HWES: 3.3 ± 0.6, p = 0.003). Complication rates, including infection, discharge, and hypertrophic scar formation, were comparable between the two methods.

Conclusion

Staple closure in orthopedic surgeries offers several advantages over sutures, including reduced operative time, less pain during removal, and superior cosmetic results. These benefits occur without an increased risk of wound complications. This study suggests that staples may be a preferable option for skin closure in orthopedic procedures, though further research with extended follow-up is needed to fully assess long-term outcomes.

## Introduction

Skin closure is an essential step in deciding the success of a surgery. Despite this, the choice of skin closure material is frequently overlooked when making surgical decisions. Surgical wound closure is crucial in restoring the tissue strength with the least possible damage and an aesthetically acceptable scar, with a low risk of complications.

Surgical site infections are one of the most dreaded and most common post-operative complications which can prolong hospital stay as well as increase the healthcare cost. Wound dehiscence is another complication which can increase the duration of stay and costs. The development of hypertrophic or keloid scars, which affect the cosmetic appearance are other complications associated with surgical wound healing, which affect patient satisfaction to a great extent. The method and material used in surgical wound closure varies with the type of surgery and the length and placement of the surgical wound. Also, surgeons tend to choose the method and material based on their past experiences and convenience.

Various methods of skin closure have been studied since the discovery of surgical interventions. Initially, eyed needles, sometimes made of bone, were used along with hemp, flax, hair, linen, pig bristles, grass reeds and other plants [1]. Sushruta described suturing with materials made of bark, tendon, hair, and silk in the year 500 BCE. Well-known surgeons such as Galen and Antyllus, Paré and Lister also described primitive sutures. One of the methods involved using pincher ants to approximate wounds, and was in vogue during the mid-twentieth century [2].

In contemporary practice, the choices for skin closure include sutures, staples, clips, skin closure strips, and topical adhesives. Each method has its own set of advantages and limitations. Sutures, while versatile and non-toxic, can be time-consuming and often result in cosmetically inferior scars. In contrast, staples offer a quicker and more efficient closure, potentially yielding better cosmetic results compared to sutures [3]. Despite the availability of various options, the fundamental question remains: sutures or staples?

Previous research has explored the comparative efficacy of sutures versus staples in various surgical contexts. For instance, studies by Gohiya et al. [3] and Ranaboldo and Rowe‐Jones [4] demonstrated that staple closures were significantly faster than sutures. Furthermore, the risk of wound infection associated with these methods has been debated, with some studies suggesting a higher risk with staples [5,6]. The cosmetic outcomes and patient satisfaction associated with each method also warrant attention, as they play a crucial role in the overall patient experience.

In the context of orthopedic surgeries, the choice of skin closure material can influence not only wound healing but also postoperative pain and patient satisfaction. Although there is substantial evidence regarding the efficacy of different closure methods, studies specifically focusing on the South Indian population are lacking. Our study aims to address this gap by comparing the outcomes of skin closure using sutures versus staples in orthopedic surgeries within a South Indian population. By evaluating closure time, pain during removal, cosmetic outcomes, and complication rates, this study aims to guide decision-making regarding the choice of closure material in orthopedic procedures, thereby enhancing patient outcomes.

## Materials and methods

All patients undergoing orthopedic procedures at Chettinad Hospital and Research Institute from July 2023 to February 2024 were screened for eligibility.

Inclusion criteria for the study were patients aged between 18 and 60 years undergoing both elective and emergency orthopedic surgeries with incisions greater than 5 cm in length. Exclusion criteria comprised individuals who did not provide consent, those with open fractures, active infections at any site, or those who were immunocompromised. Additionally, patients undergoing surgeries of the hand or foot, minimally invasive procedures, or those with incisions smaller than 5 cm were excluded. Individuals with uncontrolled systemic diseases were excluded as well. Although 154 patients were initially considered, only 120 met the eligibility criteria and were included in the study.

Patients who met the inclusion criteria and provided consent were enrolled in the study and randomized at the time of enrollment using a computer-generated randomization list. Each participant was assigned a random number, allocating them into one of two study groups. Both groups underwent identical pre-operative preparations.

The length of the surgical incision was measured, and wound closure up to the subcutaneous layer was performed with absorbable polyglactin 910 (Vicryl) sutures. Skin closure was completed using either staples or non-absorbable nylon (Ethilon) sutures, as per the randomization. The duration of the skin closure procedure was recorded.

**Figure 1:**
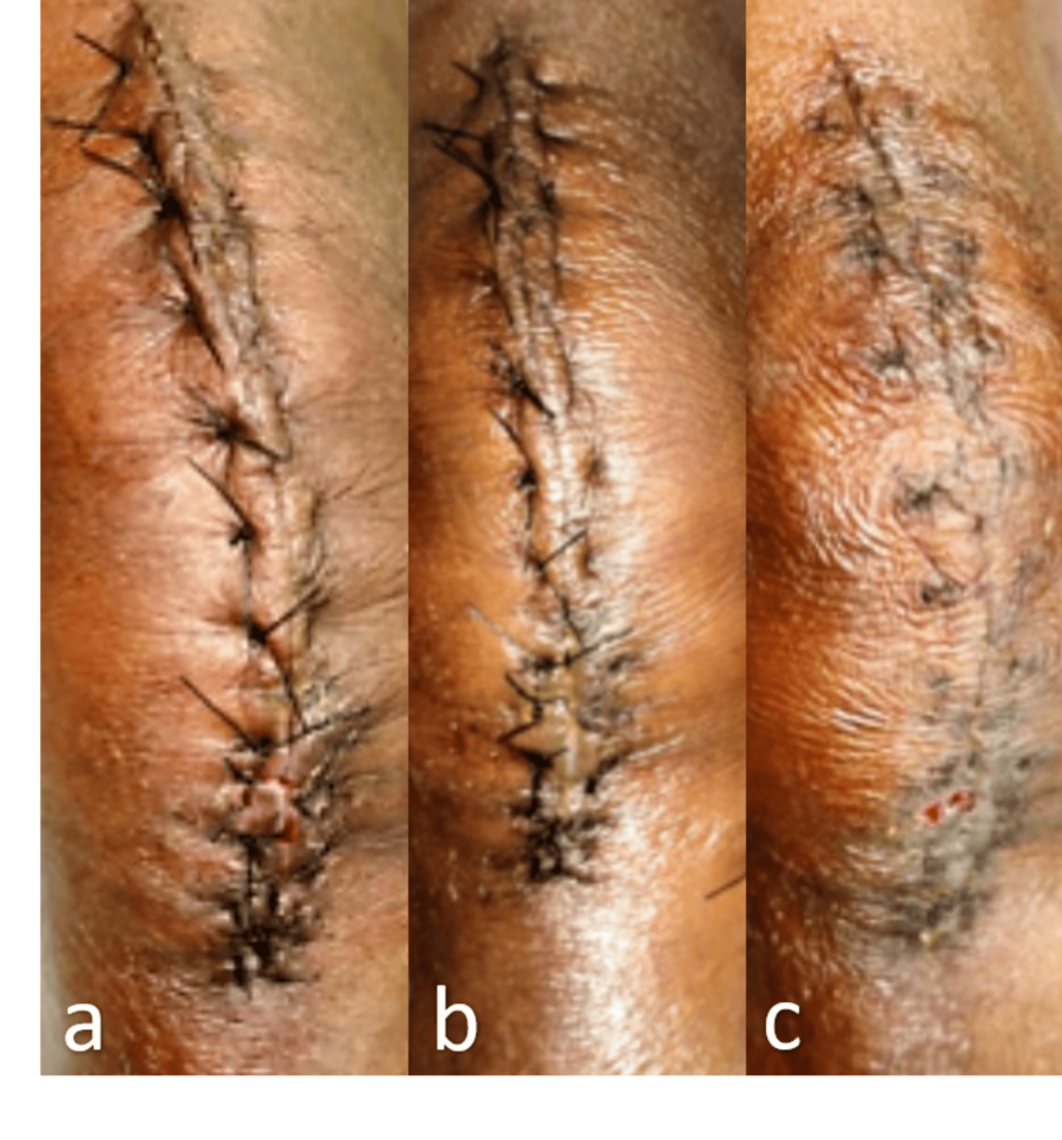
Wound closure with suture material - (a) 2nd, (b) 5th and (c) 12th post-operative days

**Figure 2:**
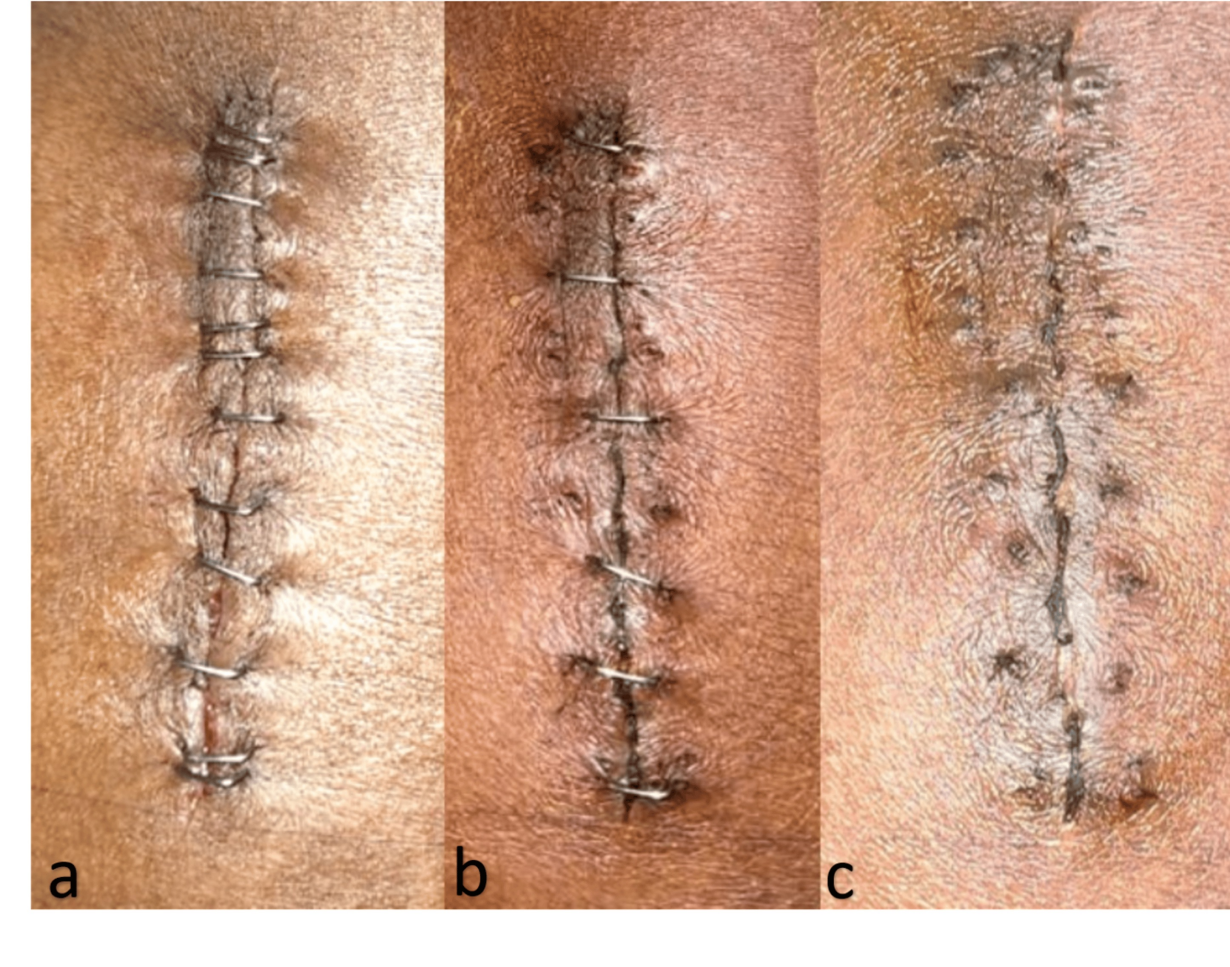
Wound closure with staples - (a) 2nd, (b) 10th and (c) 12th post-operative days

All patients received the same intravenous antibiotics until the second postoperative day. Wound inspections were conducted on the 2nd, 5th, 7th, and 12th postoperative days (Figures 1, 2). Any discharge observed during wound inspection was swabbed and sent for culture and sensitivity testing. Suture or staple removal was performed on the 12th postoperative day. Pain during removal was evaluated using the Visual Analogue Scale (VAS). Follow-up was conducted at the sixth month postoperatively, assessing cosmetic outcomes with the Hollander Wound Evaluation Score (HWES) and patient satisfaction using the VAS.

Statistical analysis was performed using IBM SPSS Statistics for Windows, Version 29.0 (IBM Corp., Armonk, NY, USA). Continuous variables, such as skin closure time, VAS, and HWES scores, were summarized as mean ± standard deviation (SD) and compared between the sutures and staples closure groups using independent two-sample t-tests. The assumption of normality was verified using the Shapiro-Wilk test, and equal variances were assessed using Levene's test.

Categorical variables, such as wound complications, infection rates, discharge from wounds, wound necrosis, and hypertrophic scar formation, were analyzed using Fisher’s Exact Test due to the relatively small sample sizes in each group. Odds ratios (OR) with 95% confidence intervals (CI) were calculated to assess the strength of association between the groups for these categorical outcomes.

A p-value of less than 0.05 was considered statistically significant for all analyses.

## Results

A total of 120 patients participated in the study, with a mean age of 41 years (range 24-58 years). The cohort included 67 males and 53 females. The patient demographics are mentioned in Table 1. A total of 28.3% (34 patients) underwent emergency surgery whereas 71.6% (86 patients) underwent elective procedures.

**Table 1:** Patient demographics

Demographic	Value
Total Patients	120
Mean Age	41 years
Age Range	24–58 years
Gender	Male (55.8%, n=67) Female (44.2%, n=53)

The mean skin closure time was significantly shorter in the staples group (5.2 ± 0.9 minutes) compared to the sutures group (12.6 ± 1.5 minutes), with a t-statistic of 15.83 and a highly significant p-value of <0.0001. This result indicates a shorter closure time with staples as compared to sutures.

The mean VAS score during removal was slightly lower in the staples group (1.41 ± 0.15) compared to the sutures group (1.57 ± 0.2), with a t-statistic of 2.39 and a p-value of 0.024. This suggests that patients in the staples group experienced less pain during removal, which was statistically significant.

The mean patient satisfaction VAS score was slightly higher in the staples group (8.35 ± 0.45) than in the sutures group (8.0 ± 0.5). However, this difference was not statistically significant (t-statistic = -1.95, p = 0.062), indicating comparable levels of patient satisfaction.

The cosmetic outcome, measured by the HWES score, was significantly better with staples (4.1 ± 0.7) than sutures (3.3 ± 0.6), with a t-statistic of -3.25 and a p-value of 0.003. This finding suggests that closure with staples may provide superior cosmetic results.

There were no statistically significant differences in the proportion of wound complications (15% in sutures vs. 8.3% in staples; OR = 3.24, 95% CI = 0.57, 18.42; p = 0.257) between the two groups. Similarly, the rates of infection (6.6% in Sutures vs. 3.3% in Staples; OR = 2.4, 95% CI = 0.37, 15.69; p = 0.648), discharge from the wound (3.3% in both groups; OR = 1.0, 95% CI = 0.14, 7.10; p = 1.000), wound necrosis (1.6% in Sutures vs. 0% in Staples; OR = Not estimable due to zero cases in staples group; p = 1.000), and hypertrophic scar formation (3.3% in Sutures vs. 1.6% in Staples; OR = 2.17, 95% CI = 0.18, 26.45; p = 1.000) were comparable. The comparative analysis of outcomes between closure with sutures and staples is presented in Table 2.

**Table 2:** Comparison of outcomes between sutures and staples (p-value < 0.05 considered significant) VAS: Visual Analog Scale; HWES: Hollander Wound Evaluation Score

Outcome	Group	Mean ± SD / % (n)	t-statistic / OR (95% CI)	p-value
Mean skin closure time (min)	Sutures	12.6 ± 1.5	15.83	<0.0001
	Staples	5.2 ± 0.9		
Mean VAS score during removal	Sutures	1.57 ± 0.2	2.39	0.024
	Staples	1.41 ± 0.15		
Mean patient satisfaction VAS score	Sutures	8.0 ± 0.5	-1.95	0.062
	Staples	8.35 ± 0.45		
HWES score (cosmesis)	Sutures	3.3 ± 0.6	-3.25	0.003
	Staples	4.1 ± 0.7		
Wound complications	Sutures	15% (n=9)	3.24 (0.57, 18.42)	0.257
	Staples	8.3% (n=5)		
Infection	Sutures	6.6% (n=4)	2.4 (0.37, 15.69)	0.648
	Staples	3.3% (n=2)		
Discharge from wound	Sutures	3.3% (n=2)	1.0 (0.14, 7.10)	1.000
	Staples	3.3% (n=2)		
Wound necrosis	Sutures	1.6% (n=1)	Not estimable	1.000
	Staples	0% (n=0)		
Hypertrophic scar formation	Sutures	3.3% (n=2)	2.17 (0.18, 26.45)	1.000
	Staples	1.6% (n=1)		

## Discussion

The choice of skin closure material is an afterthought during orthopedic surgeries as the choice of implant or fixation method takes precedence. Although often overlooked, the choice of material for skin closure is equally important as it can impact the rate of surgical site infections. The current study aimed to guide surgeons to choose a suitable wound closure material by comparing sutures and staples in the South Indian population.

Previous studies, such as those by Gohiya et al. [3] and Ranaboldo and Rowe‐Jones [4], have demonstrated that closure with staples is significantly faster than with sutures. Gohiya et al. [3] reported that closure with staples was 4.42 times faster than suturing with nylon, and Ranaboldo and Rowe‐Jones [4] found that the rate of closure with staples was significantly higher than with sutures (P < 0.0002). Consistent with these findings, our study showed a significant difference in skin closure time, with a mean duration of 5.2 minutes for staples and 12.6 minutes for sutures (p < 0.0001). This 7.4-minute reduction in surgical time when using staples highlights their efficiency and potential benefits in reducing the operative time.

The incidence of wound discharge has been debated in the literature, with mixed findings. A meta-analysis by Smith et al. [5] found no significant difference in the incidence of wound discharge between sutures and staples in the closure of hip surgeries. On the other hand, Singh et al. [6] reported a statistically higher incidence of wound discharge with staples than with sutures. Gohiya et al. [3] found that the wound discharge was maximum on 2nd post-operative day. Our study showed no change in the incidence of post-operative wound discharge with staples and sutures (3.3%, n=2 in both groups).

A meta-analysis conducted by Smith et al. [5] found that the risk of wound infection was four times greater in those cases where the wounds were closed with staples than with sutures. Gohiya et al. [3] observed a higher rate of infection in the staples group than the suture group, which was statistically significant. Fewer wound infections were seen with sutures in a study by Cochetti et al. [7]. Contrary to these findings, our study observed a lower rate of infection in the staples group (3.3%) compared to the sutures group (6.6%), although this difference was not statistically significant (p = 0.648). This could be attributed to the design of staples, which may reduce the introduction of skin flora into the wound, as noted by Johnson et al. [8]. With conventional suturing, the needle carries the epidermis and organisms along its track and into the depths of the wound, thereby posing a higher risk for infection. Also, suture materials can harbor bacterial biofilm formation [9], even though all suture materials are not necessarily equivalent in this regard. The conduction of surface bacteria by percutaneous multifilament sutures by wicking effect can also be a reason for infection [10].

Gatt et al. [11] reported no difference in difficulty or pain during the removal of staples compared to sutures. However, our study found that patients in the staples group experienced significantly less pain during removal, with a VAS score of 1.41 compared to 1.57 in the sutures group (p = 0.024). This suggests that staples may be a more comfortable option for patients, potentially improving the overall surgical experience.

Clayer and Southwood [12] reported the scars of closure with sutures to be thinner than those with staples. Gatt et al. [11] and George [13] found no significant statistic difference in the cosmesis between suture and staple closure. Khan et al. [14] found the HWES score to be 5.3 in the staple group and 6 in the suture group. In our study, the HWES score was significantly higher in the staples group (4.1 ± 0.7) compared to the sutures group (3.3 ± 0.6, p = 0.003), indicating a cosmetically superior scar with staple closure.

The incidence of hypertrophic scars and keloids has been reported to be lower with staples in previous studies [15, 16]. Our study supports these findings, showing a lower incidence of hypertrophic scar formation with staples (1.6%) compared to sutures (3.3%), though this difference was not statistically significant (p = 1.000). This may be due to the better opposition of wound edges achieved with staples, which minimizes tension across the wound and reduces the likelihood of keloid formation.

Batra et al. [17] found wound dehiscence to be more pronounced in staple wound closure compared to sutures. According to a meta-analysis of closure methods in caesarean delivery by Clay et al. [18], the rate of wound dehiscence was significantly higher in staple closure with respect to sutures. These findings could be due to the different inclusion criteria and methodological limitations.

This study demonstrates that the use of staples for skin closure in orthopedic surgeries offers several advantages over sutures, including reduced operative time, lower post-operative pain, and potentially better cosmetic outcomes, without an increased risk of wound complications. It is also important to note that the cost of sutures is lower than that of staples, which could be valuable in resource-limited settings. Despite having a higher initial cost as compared to sutures, the potential reduction in the operative time, duration of hospital stay and complications, when using staples can offset some of these expenses, making them a cost-effective option in the long run.

However, the study's follow-up period was limited to six months, which may underestimate the true incidence of long-term wound complications. Further studies with longer follow-up periods are recommended to validate these findings. The single-center design could limit generalizability. The sample size for certain outcomes (e.g., wound complications, infections) was relatively small. This small sample size may affect the statistical power to detect differences in less common complications. Larger sample sizes in future studies could provide more robust data.

## Conclusions

In this comparative analysis of sutures versus staples for skin closure in orthopedic surgeries, staples demonstrated notable advantages, including a significantly shorter closure time and less pain during removal. Staples provided better cosmetic outcomes without increasing the risk of complications such as infection or hypertrophic scarring. While patient satisfaction remained comparable across both methods, the efficiency and aesthetic benefits of staples emphasize their potential superiority in surgical skin closure. Future studies with longer follow-up are warranted to confirm these findings and explore long-term implications.
